# Asthma Prevalence Associated with Geographical Latitude and Regional Insolation in the United States of America and Australia

**DOI:** 10.1371/journal.pone.0018492

**Published:** 2011-04-08

**Authors:** Goran Krstić

**Affiliations:** Fraser Health, Environmental Health Services, New Westminster, Canada; University of North Carolina at Chapel Hill, United States of America

## Abstract

**Background:**

It has been proposed that vitamin D deficiency may be responsible for an increase in the prevalence of allergic diseases and asthma worldwide. Human ability to generate physiologically required quantities of vitamin D through sun exposure is decreasing with increasing geographical latitude.

**Objectives:**

Considering that vitamin D deficiency is usually due to lack of outdoor sun exposure, this study is designed to test the hypothesis that a higher prevalence of asthma should be expected at high relative to low geographical latitudes.

**Methods:**

Linear regression analyses are performed on asthma prevalence in the U.S. adult population vs. geographical latitude, insolation, air temperature, and air pollution (PM_2.5_) for 97 major metropolitan/micropolitan statistical areas of the continental United States of America and on general population asthma prevalence vs. geographical latitude in eight metropolitan areas of Australia.

**Results:**

A 10° change in geographical latitude from southern to northern regions of the Eastern Seaboard is associated with a 2% increase in adult asthma prevalence (*p*<0.001). Total insolation in winter months is almost as strong as latitude in its ability to explain the observed spatial variation in the prevalence of asthma (r^2^ = 0.43; *p*<0.001). Similar results are obtained using the Australian data (r^2^ = 0.73; p<0.01), suggesting a consistent association between the latitude/insolation and asthma prevalence worldwide.

**Conclusions:**

The results of this study suggest that, as a known modulator of the immune response closely linked with the geographical latitude and erythemal UV irradiation, vitamin D may play an important role in the development/exacerbation of asthma.

## Introduction

Both positive [1], [2] and negative associations [3], [4] between the prevalence of asthma/allergies and geographical latitude have been reported in the published literature. This is an ecological study designed to test the proposed hypothesis that the prevalence of asthma increases with increasing latitude due to a decreasing intensity of solar irradiation which effectively reduces the individual's cutaneous generation of vitamin D.

Maternal vitamin D deficiency/insufficiency is associated with an increased probability of developing asthma and allergy-related symptoms in early life of their offspring. Camargo, Devereux and colleagues concluded that specifically in the northeastern United States of America, an increased maternal intake of vitamin D from diet or supplements during pregnancy may decrease the risk of wheeze symptoms in early childhood [5], [6]. Low levels of serum vitamin D in adults have been associated with impaired lung function, increased airway hyperresponsiveness, and reduced glucocorticoid response in asthma [7]. Litonjua and Weiss proposed that vitamin D deficiency may be responsible for an increase in the prevalence of allergic diseases and asthma worldwide, as more time is spent indoors with less exposure to sunlight, leading to a decreased cutaneous vitamin D production [8]. Exposure to solar ultraviolet radiation within a wavelength band of 290–315 nm (UV-B) and production of vitamin D in the skin is the primary source of vitamin D for many people [9], particularly for those who do not receive adequate vitamin D doses through diet.

Higher rates of emergency department visits for acute allergic reactions have been observed in northeastern when compared to southern regions of the United States [10], suggesting an association with latitudinal difference in insolation. Kimlin and colleagues observed that the available levels of erythemal or vitamin D producing UV-B irradiation decreases dramatically as the latitude increases [11], particularly during the four cooler months (i.e., November to February). The efficiency of vitamin D production in the exposed skin depends on the dose of solar UV-B radiation, which can be curtailed by clothing, excess body fat, sunscreen, and the skin pigment melanin [12]. Higher prevalence of vitamin D deficiency has been found among inner-city African American youth with asthma when compared to non-asthmatic control subjects [13].

An intense annual insolation in the lower latitudes of the southern U.S. regions provides greater potential for the cutaneous vitamin D production in exposed individuals. It is estimated that over 95% of the variability in average daily UV dosages can be explained by the latitude and altitude, where the effect of the latitude on the UV irradiation on the Earth's surface is much more significant than the altitude. The longitude is not statistically significant in terms of its ability to predict the intensity of the UV irradiance [14]. Considering that vitamin D is suspected to have a role in asthma development [8], [15] and that vitamin D deficiency is usually due to lack of outdoor sun exposure [11], [12], it is hypothesized in this study that a higher prevalence of asthma should be expected at high relative to low geographical latitudes.

## Materials and Methods

### Asthma – U.S. data

The data on asthma prevalence in the U.S. adult population (i.e., age 18 and over) is obtained from the U.S. Department of Health and Human Services (DHHS), Centers for Disease Control and Prevention (CDC) Behavioral Risk Factor Surveillance System [16]. The mean annual asthma prevalence in U.S. “*adults who have been told they currently have asthma*” for the period from 2006 to 2008 by Metropolitan/Micropolitan Statistical Area (MMSA) are included in this study. The obtained data relates to simple prevalence by MMSA and contains no patient-specific information. Therefore, there was no need to request an ethics committee approval or a written consent from the patients. All asthma prevalence data used in this study are freely available to download from online resources. The citation recommended by the CDC is used to reference the source of asthma prevalence information for this research paper.

A continuity of geographical latitude and longitude is considered as important for reducing the influence of statistical outliers and implementing a meaningful regression analysis. Hence, the studied U.S. geographical area includes all continental states with the exception of Alaska. Asthma prevalence data are matched with the data on Air Quality Trends by Core Based Statistical Areas (CBSA), obtained from the U.S. Environmental Protection Agency (EPA) [17]. The resulting 97 matched and collated U.S. wide MMSAs/CBSAs are used in the statistical analyses presented in this paper.

### Asthma – Australian data

The Australian general population asthma prevalence data by Local Government Area (LGA) for the period from 2004 to 2005 are obtained from the publically available online resource of the Australian Public Health Information Development Unit [18]. The studied metropolitan areas of Australia include: Sydney – New South Wales (NSW), Melbourne – Victoria (Vic), Brisbane – Queensland (Qld), Adelaide – South Australia (SA), Perth – Western Australia (WA), Hobart – Tasmania (Tas), Darwin – Northern Territory (NT), and Canberra – Australian Capital Territory (ACT).

### Geographical coordinates data

Geographical latitudes and longitudes for the main cities in the MMSAs are obtained using Microsoft Research (MSR) Maps [19]. The geographical information and maps made available online by the MSR are supplied through their partnership with the U.S. Geological Survey (USGS). The geographical coordinates for Australian metropolitan areas are obtained from the Guide to Australia at Charles Sturt University online resource, which is based on the 1996 data from the Australian Bureau of Statistics [20].

### Air pollution and meteorological data

The air pollution data for fine airborne particulate matter, aerodynamic diameter of less than 2.5 µm (i.e., PM_2.5_), are obtained from the U.S. EPA Air Trends online information resource [17]. Mean annual PM_2.5_ concentrations expressed in µg/m^3^ for the period from 1999 to 2008 by matched CBSAs/MMSAs are included in this study.

The mean annual insolation on horizontal surface and air temperature, expressed in kWh/m^2^/day and °C, respectively, for the MMSAs/CBSAs in the period from 1983 to 2005 are obtained from the National Aeronautics and Space Administration (NASA) Atmospheric Science Data Center [21]. Central latitudes and longitudes of cities/statistical areas, as presented by the USGS, are used to obtain the data on annual mean insolation and air temperature at specific geographical coordinates and regional elevation.

### Statistical analysis

Descriptive statistics and scatter plot analysis revealed no evidence of non-normality in the distribution of asthma prevalence, geographical coordinates, insolation, air temperature, and air pollution data, allowing the use of linear regression analysis models to evaluate the relationships between the studied variables.

Linear regression and correlation analyses are implemented to evaluate the strength, direction, and statistical significance of regression/correlation coefficients. The selected independent variables (i.e., regional horizontal surface insolation, latitude, air temperature, and air pollution) are compared in terms of their ability to explain the observed variation in the dependent/response variable, the prevalence of asthma in the U.S. adult population, by calculating the coefficients of determination (***r^2^***). Linear regression equations are developed and tested for their ability to predict the prevalence of asthma in 97 major metropolitan/micropolitan areas of the continental U.S. and a subset of 39 areas from the Eastern Seaboard in response to meteorology and air pollution at different latitudes. The same approach is applied to test the correlation between the general population asthma prevalence and the geographical latitude in eight metropolitan areas of Australia.

## Results

**Figure 1:**
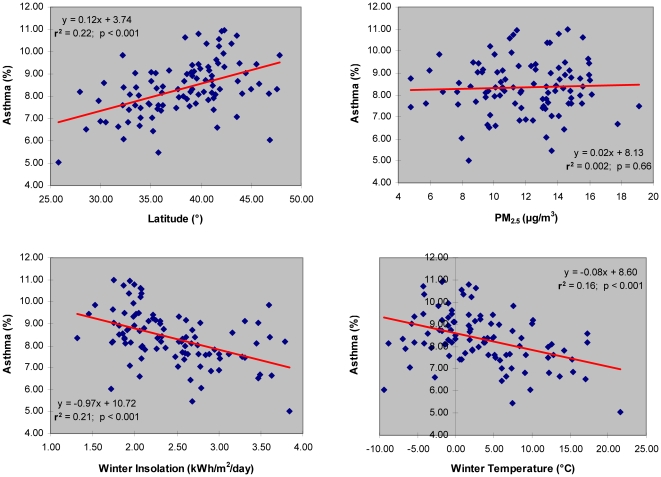
Asthma prevalence vs. latitude, air pollution (PM_2.5_), winter insolation, and winter temperature in adult population of 97 major metropolitan/micropolitan areas of continental U.S.

The prevalence of asthma in the U.S. adult population, as presented in **Table 1** and **Figure 1**, is associated with geographical latitude (r^2^ = 0.22; *p*<0.001), annual mean insolation on horizontal surface (r^2^ = 0.15; *p*<0.001), and annual regional air temperature (r^2^ = 0.17; *p*<0.001). The association of asthma prevalence with the annual mean air pollution as PM_2.5_ is very weak and not statistically significant (r^2^ = 0.002; *p* = 0.66). Although the annual air temperature appears to be a marginally better predictor of asthma prevalence than the annual mean insolation in the studied population, both insolation (r^2^ = 0.48) and air temperature (r^2^ = 0.84) are correlated with the geographical latitude (**Table 1**).

**Table 1:** Linear regression estimates of asthma prevalence in US adult population associated with latitude, annual mean insolation, air temperature and air pollution (PM_2.5_).

Linear Regression Coefficient/Variable‡	Model 1 - Asthma vs. Latitude	Model 2 - Asthma vs. Insolation	Model 3 - Asthma vs. Temperature	Model 4 - Asthma vs. PM_2.5_	Model 5 - Insolation vs. Latitude	Model 6 - Temperature vs. Latitude
Y-axis Intercept	3.74±0.90***	12.22±0.96***	9.77±0.34***	8.13±0.52***	7.08±0.31***	45.74±1.50***
Latitude (°)	0.12±0.02***	—	—	—	−0.07±0.01***	−0.87±0.04***
Annual Mean Insolation (kWh/m^2^/day)	—	−0.92±0.23***	—	—	—	—
Annual Mean Air Temperature (°C)	—	—	−0.11±0.03***	—	—	—
Air Pollution (PM_2.5_) (µg/m^3^)	—	—	—	0.02±0.04	—	—
Number of Areas	97	97	97	97	97	97
Coefficient of Determination (r^2^)	**0.22**	**0.15**	**0.17**	**0.002**	**0.48**	**0.84**

***p<0.001.

‡ Plus-minus values are linear regression coefficients and standard errors (i.e., ±SE).

The best predictor of asthma prevalence among the studied variables is the geographical latitude with an ability to explain up to 22% of the variation in the prevalence of asthma in the continental U.S. adult population. Based on the linear regression analysis, a 10° change in the geographical latitude from southern to northern U.S. regions is associated with a 1.2% increase in the prevalence of adult asthma. The highest prevalence of adult asthma is observed in Detroit, MI at 10.97% (latitude: 42.35° North) and the lowest in Miami, FL at 5.03% (latitude: 25.81° North).

Bothe the regional insolation on horizontal surface (r^2^ = 0.21; *p*<0.001) and air temperature (r^2^ = 0.16; *p*<0.001) in winter months (i.e., November to February) are statistically significant predictors of asthma prevalence (**Table 2** **,** **Figure 1**). However, regional insolation during winter appears to be almost as effective as latitude in terms of its ability to predict the prevalence of asthma in the U.S. adult population. When compared to the results of regression analyses using annual mean values, a stronger association between latitude and insolation (r^2^ = 0.80; *p*<0.001) is observed for winter months, which is in agreement with the findings of Kimlin and colleagues [11].

**Table 2:** Linear regression estimates of asthma prevalence in US adult population associated with insolation and air temperature in winter months (November to February).

Linear Regression Coefficient/Variable‡	Model 7 - Asthma vs. Insolation	Model 8 - Asthma vs. Temperature	Model 9 - Insolation vs. Latitude	Model 10 - Temperature vs. Latitude
Y-axis Intercept	10.72±0.48***	8.60±0.13***	6.64±0.21***	48.68±2.61***
Latitude (°)	—	—	−0.11±0.01***	−1.19±0.07***
Winter Mean Insolation (kWh/m^2^/day)	−0.97±0.19***	—	—	—
Winter Mean Air Temperature (°C)	—	−0.08±0.02***	—	—
Number of Areas	97	97	97	97
Coefficient of Determination (r^2^)	**0.21**	**0.16**	**0.80**	**0.76**

***p<0.001.

‡ Plus-minus values are linear regression coefficients and standard errors (i.e., ±SE).

The correlation matrix in **Table 3** shows that asthma prevalence is best explained by the variation in geographical latitude (r = 0.47) and winter insolation (r = −0.46), closely followed by annual air temperature (r = −0.42). Both the geographical longitude (r = 0.15) and air pollution (r = 0.04) showed weak and not statistically significant correlation with the prevalence of asthma in U.S. adult population. Latitude is best correlated with annual air temperature (r = −0.92), closely followed by winter insolation (r = −0.90).

**Table 3:** Correlation matrix for asthma prevalence in US adult population, latitude, longitude, air pollution (PM_2.5_), annual insolation, winter insolation, annual air temperature, and winter air temperature.

	Latitude (°)	Longitude (°)	Air Pollution (PM_2.5_) (µg/m^3^)	Annual Insolation (kWh/m^2^/day)	Winter Insolation (kWh/m^2^/day)	Annual Temperature (°C)	Winter Temperature (°C)	Asthma (%)
**Latitude (°)**	1.00							
**Longitude (°)**	−0.04	1.00						
**Air Pollution (PM_2.5_) (µg/m^3^)**	−0.15	0.43	1.00					
**Annual Insolation (kWh/m^2^/day)**	−0.69	−0.54	−0.29	1.00				
**Winter Insolation (kWh/m^2^/day)**	−0.90	−0.24	−0.13	0.91	1.00			
**Annual Temperature (°C)**	−0.92	0.13	0.29	0.51	0.75	1.00		
**Winter Temperature (°C)**	−0.87	0.04	0.24	0.51	0.73	0.97	1.00	
**Asthma (%)**	0.47	0.15	0.04	−0.38	−0.46	−0.42	−0.40	1.00

**Figure 2:**
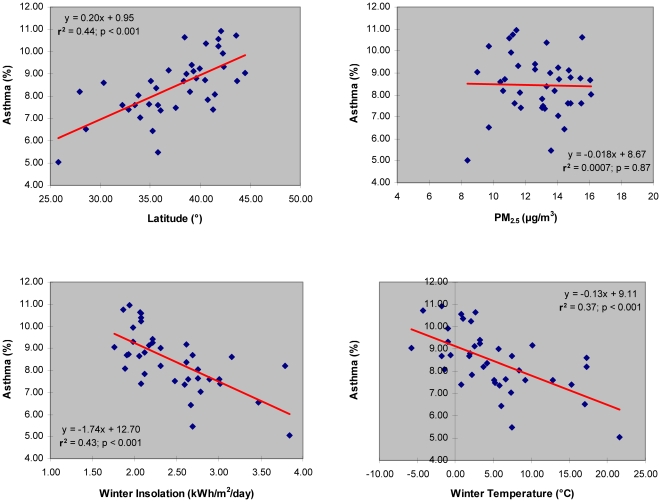
Asthma prevalence vs. latitude, air pollution (PM_2.5_), winter insolation, and winter temperature in adult population of 39 major metropolitan/micropolitan areas of the eastern seaboard.

The regression analysis of a subset of data covering 39 major metropolitan/micropolitan areas in the U.S. regions of the Eastern Seaboard (i.e., New England, Middle Atlantic, and South Atlantic) shows a very strong association between asthma prevalence and winter insolation (r^2^ = 0.43; *p*<0.001). A 10° change in the geographical latitude from southern to northern regions of the Eastern Seaboard is associated with a 2% change in adult asthma prevalence (**Figure 2**). Considering that asthma prevalence estimates are more reliable for the U.S. states and regions with larger population size, having narrower 95% confidence intervals [16], and that the Eastern Seaboard is home to most highly populated U.S. cities, a regression analysis based on the data from this region is expected to be more reliable when compared to the data for all continental U.S. regions.

Multiple regression analyses are performed on asthma prevalence vs. insolation, air temperature, and air pollution in an attempt to identify independent effects of the studied predictors (**Table 4**). Air temperature and insolation are the only statistically significant predictors of asthma in the continental U.S., where the air temperature appears to be the best predictor based on total annual data (annual mean air temperature: *p*<0.01; annual mean insolation: *p* = 0.17) and the insolation when only winter data is applied (winter mean insolation: *p*<0.05; winter mean air temperature: *p* = 0.31). Air pollution is not statistically significant predictor of asthma in any of the models.

**Table 4:** Multiple regression estimates of asthma prevalence in US adult population associated with annual and winter mean insolation, annual and winter mean air temperature and air pollution (PM_2.5_).

Multiple Regression Coefficient/Variable‡	Model 11 - Asthma vs. Annual Mean Insolation, Temperature, and PM_2.5_			Model 12 - Asthma vs. Winter Mean Insolation, Temperature, and PM_2.5_		
		Collinearity Statistics			Collinearity Statistics	
		Tolerance	VIF#		Tolerance	VIF#
Y-axis Intercept	10.86±1.38***			10.01±1.06***		
Annual Mean Insolation (kWh/m^2^/day)	−0.42±0.30	0.53	1.89	—		
Annual Mean Air Temperature (°C)	−0.10±0.03**	0.53	1.89	—		
Winter Mean Insolation (kWh/m^2^/day)	—			−0.72±0.32*	0.36	2.75
Winter Mean Air Temperature (°C)	—			−0.03±0.03	0.35	2.87
Air Pollution (PM_2.5_) (µg/m^3^)	0.04±0.05	0.65	1.53	0.02±0.04	0.74	1.36
Number of Areas	97			97		
Coefficient of Determination (R^2^)	**0.22**			**0.22**		
Adjusted R^2^	**0.19**			**0.20**		

***p<0.001;

**p<0.01;

*p<0.05.

‡ Plus-minus values are multiple regression coefficients and standard errors (i.e., ±SE).

# VIF – Variance Inflation Factor.

**Figure 3:**
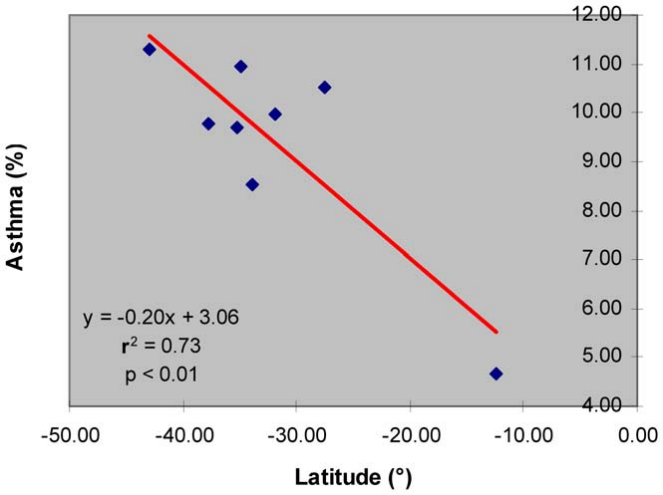
Asthma prevalence vs. latitude in the population of 8 major metropolitan areas of Australia.

The observed correlation between asthma prevalence and absolute values of geographical latitude for eight Australian metropolitan regions is positive, statistically significant, and in agreement with the results obtained using the U.S. adult asthma prevalence data (**Figure 3**). The results show that up to 73% of the variation in asthma prevalence in the Australian general population could be explained by the variation in the geographical latitude (r^2^ = 0.73; p<0.01). A 10° change in the geographical latitude from the North to the South is associated with approximately the same increase in asthma prevalence as presented in **Figure 2** for the U.S. Eastern Seaboard region from the South to the North (i.e., ∼2%). It is important to observe that latitudes in the southern hemisphere have negative values and that, although the relationship between the absolute latitude and asthma is positive, the correlation coefficient is mathematically negative.

## Discussion

There are pros as well as cons associated with study designs in epidemiological research. An ecological study design, as presented in this and similar papers, has some known limitations in terms of its ability to provide reliable inferences about the population characteristics at individual level. As discussed by Robinson in 1950, ecological correlations cannot be validly used as substitutes for individual correlations [22]. Using the same principle one could argue that the opposite also applies, where one cannot make valid inferences about large populations and their possible interactions by focusing only on the individuals from those populations. Robinson indicated that the purpose of his paper is to prevent “*the future computation of meaningless correlations*”. However, despite lacking the ability to deal with health risk factors at a small-scale individual level, well designed ecological studies provide meaningful correlations and inferences that are useful when dealing with national and international public health issues and health risk factors at a large-scale population level. In their revisiting of Robinson's paper, Subramanian et al. [23] concluded that “*… perils are posed by not only ecological fallacy but also individualistic fallacy*”.

Although a similar study design was applied by Staples and colleagues [3], this is the first comprehensive study that includes the populations of the continental United States and Australia, covering over 100 metropolitan areas within a substantial latitudinal range from both the northern and the southern hemispheres. It is interesting that Staples and colleagues observed a negative correlation between the prevalence of asthma and latitude, which is in contrast to the results of the study on the basis of more recent data for both the U.S. and Australia as presented in this paper. It should be taken into consideration that Staples et al. (2003) study was based on the 1995 Australian National Health Survey of approximately 54,000 people from all states and territories and across all age groups. In addition, asthma prevalence data were based on state/territory while geographical latitudes represented smaller areas of corresponding capital regions. The authors indicated that the latitude ranges for some states/territories spanned over 10° (up to 19° for Queensland). Yet a single latitude coordinate was applied for each of the states/territories, not necessarily representing the areas from which the cases of asthma were identified and clearly affecting the accuracy of the observed relationship between the asthma prevalence and geographical latitude in Australia.

Franco et al. [1] showed a statistically significant positive correlation between geographical latitude and active asthma prevalence in the eight International Study of Asthma and Allergies in Childhood (ISAAC) centres in North-East Brazil. The authors found no relation between the tropical weather and high prevalence of childhood asthma in the studied population.

Weiland et al. [2] studied the association between climate and atopic diseases using the data from 146 ISAAC centres worldwide and found that the prevalence of eczema symptoms was positively associated with latitude. The authors observed a similar association in both 6–7 and 13–14 years age-groups in Europe and also among 6–7 years old children worldwide, suggesting that “… *latitude and temperature affect the prevalence of eczema only indirectly, due to changes in behaviour and differences in sun exposure*” (emphasis added). They concluded that “*climate may affect the prevalence of asthma and atopic eczema in children*”.

In an ecological analysis of geo-climatic variations in the prevalence of current asthma, allergic rhinitis and chronic cough, and phlegm in Italy, Zanolin et al. [4] found a negative correlation between asthma-like symptoms and geographical latitude. Rather than to focus specifically on the diagnosed asthma cases country-wide, the study is based on a random sample of 18,873 subjects with a response rate of 72.7% from different climatic regions, effectively representing less than 0.03% of the Italian total general population. The authors suggested that “*variations in the prevalence of respiratory symptoms according to geo-climatic factors could provide important clues to the knowledge of the aetiology of asthma*”.

A report from the US CDC on The State of Childhood Asthma, United States, 1980–2005 [24] indicates that current asthma prevalence rates among children 0–17 years of age, by state, annual average for the period 2001–2005 are generally higher in the northeast region of the United States. These findings are in agreement with the results and observations for U.S. adult population, by metropolitan/micropolitan statistical area, presented in this paper.

Ethnic groups with darker skin have higher prevalence of asthma when compared to those with lighter skin pigmentation [13], [25], [26], [27]. Darker skin pigmentation is a form of evolutionary adaptation, providing protection against potentially harmful effects of excessive UV irradiation doses, developed in populations inhabiting tropical regions [28]. Therefore, when compared to natives of higher latitudes with lighter skin pigmentation, natives of tropical regions migrating from lower to higher latitudes may become disadvantaged in terms of their ability to synthesize physiologically required quantities of vitamin D under low levels of annual/winter solar irradiation [29], [30]. This may result in a potentially severe deficiency if sufficient doses of vitamin D are not obtained through diet and/or supplementation.

Different immunologic, genetic and environmental mechanisms are considered in the etiology of asthma and it is proposed that geographical variation in asthma prevalence could be due to gene-by-environment interactions [31]. Although cold air has been associated with worsened respiratory symptoms and exacerbation of asthma, ambient air temperature is expected to be a symptom trigger rather than a causal factor initiating respiratory diseases [32]. Asthma attacks can be triggered by cold air, irritating fumes, or fine airborne particulate matter [33].

Vitamin D has been recognized as an important immuno-modulating factor with dendritic cells as its primary targets [34]. Calcitriol (1,25-dihydroxycholecalciferol), the main vitamin D metabolite, inhibits dendritic cell maturation and T-helper1 (Th1) cell differentiation, which has been described as a key mechanism of allergy development [35]. The inhibition of Th1 cell differentiation leads to a predominance of Th2 cells, which has been implicated in asthma pathogenesis [36], [37]. Wjst and Dold (1999) proposed that nutritional intake of vitamin D for rickets prophylaxis could be responsible for the increase in the prevalence of asthma in developed countries over the last three decades, suggesting that if protective antigen-reactive Th1 memory cells fail to develop the subsequent predominance of Th2 cells can trigger allergic reactions [38].

However, the mechanism of action of vitamin D and its active metabolites is quite complex and not yet fully understood. One should take into consideration that, in addition to the inhibition of dendritic cell maturation, activated vitamin D modulates the immune response through inhibition or enhancement on multiple levels of cell function, such as production of both pro- and anti-inflammatory cytokines, and inhibition of B cell differentiation, proliferation, and antibody secretion [34]. Activated vitamin D enhances the development of interleukin-IL-10- and reduces the number of IL-6- and IL-17-secreting cells [39]. High levels of pro-inflammatory IL-6 and IL-17 cytokines [40], [41], [42] and low levels of an anti-inflammatory cytokine IL-10 have been observed in asthmatic patients [43], [44], suggesting that a functional vitamin D insufficiency/deficiency may be responsible for an increased probability of developing asthma. Allergies and asthma could be the result of unbalanced metabolic transformation or inadequate vitamin D receptor (VDR) binding, leading to plasma accumulation of active/inactive vitamin D metabolites, predominance of pro-inflammatory cytokines and an impaired immuno-modulation.

The observed correlation between geographical longitude and annual insolation (r = −0.54) presented in **Table 3** is in agreement with the estimates of monthly mean erythemal UV irradiation values for the U.S. and Canada [45], showing an increase of solar irradiation in the direction from northeastern to southwestern regions of North America. Considering that total insolation is almost perfectly correlated with the UV component of the total solar irradiation on the Earth's surface (r>0.96, [46], [47]), the correlation between latitude and total insolation presented in this paper could be used to predict the effect of erythemal UV irradiation on the skin production of vitamin D in the studied population.

The results of linear regression analyses performed in this study provide evidence that the geographical latitude can be used to predict the prevalence of asthma in the U.S. adult population. It is interesting that in winter months the association of asthma prevalence with air temperature is weaker than the association with insolation. If the factor associated with geographical latitude and asthma prevalence is air temperature and not insolation, one would expect cold weather in winter months to yield a stronger correlation of asthma prevalence with air temperature than insolation. A contrary is observed in this study, where winter insolation is almost as good as geographical latitude in terms of its ability to predict the prevalence of asthma. In addition, the results of multiple regression analyses presented in **Table 4** confirm that, based on the data for winter insolation, winter air temperature, and air pollution, insolation is the best predictor of asthma.

Winter insolation is consistently a stronger predictor of asthma prevalence than winter air temperature in both the continental U.S. population and a subset of data for the Eastern Seaboard states. It appears that populations living in northern regions may have wider swings in vitamin D levels due to a significant decrease in vitamin D production during the winter months. When compared to those living in tropical areas, populations in the northern regions have much lower production of vitamin D in summer months which may lead to a weak physiological baseline at the beginning of winter. This condition could then progress into a vitamin D insufficiency or a severe deficiency in winter months, when regional insolation is at its annual minimum, potentially affecting the immune system and increasing the probability of respiratory infection and developing/exacerbating asthma.

The results of statistical analyses presented in this paper indicate that the probability for the observed association between latitude/insolation and asthma prevalence to be simply due to a chance is very small (i.e., p<0.01). In addition, consistent results are obtained using the data from the U.S. and Australia further reducing the probability that this may be just a property of the data distribution and not a biological or a physiological response.

Camargo et al. (2011) studied the relationship between cord-blood levels of an active metabolite of vitamin D, 25-hydroxyvitamin D (25[OH]D), and the risk of respiratory infection, wheezing, and asthma [48]. The authors concluded that cord-blood levels of 25[OH]D are inversely associated with the risk of respiratory infection and childhood wheezing but no association with incident asthma is observed. These results suggest that active metabolites of vitamin D could have an effect on reducing the frequency of respiratory infections, which may lead to a reduction in exacerbation of symptoms in those suffering from asthma.

Allan et al. (2010) conducted a case-control study of vitamin D status and asthma in 160 adults aged between 15 and 80 years, 80 with physician-confirmed mild/moderate asthma and 80 age and gender-matched controls who had a smoking history of <10 pack-years [49]. The majority of controls (i.e., 70%) were recruited from local surgery units and the remainder through the advertising in local press. The study showed no significant difference in the serum 25-hydroxyvitamin D3 concentrations between cases and controls, and no association between 25-hydroxyvitamin D3 levels and asthma severity or lung function. The authors concluded that this study does not find evidence to support the use of vitamin D as an adjunct to conventional therapy in asthma in adults. However, this study is based on a rather small group of individuals who were confined to a small geographical area with only a snapshot in time for a vitamin D status. Although high doses of vitamin D may not be useful in the treatment of adult asthma as an existing condition, the study by Allan et al. does not address possible effects of a long-term vitamin D deficiency/insufficiency, an impaired immuno-modulation, and how these parameters may affect the prevalence of asthma in large populations and over large geographical areas with significantly different levels of annual UV-B insolation.

Air temperature and confounding factors that could be associated with the geographical latitude, such as socio-economic status, regional diet, demographic structure and ethnic origin, or some unknown effects of insolation and UV radiation cannot be eliminated based on the results of this study. However, in conjunction with other published studies suggesting a possible link between vitamin D and asthma, a significant decrease of vitamin D producing erythemal UV irradiation with an increase in latitude provides a plausible explanation for the observed geographical distribution of asthma prevalence in both the northern and the southern hemispheres. As a modulator of the immune response, vitamin D could have an influence on the frequency and severity of respiratory infections which may lead to exacerbation of symptoms from preexisting asthma. A U.S.-wide comprehensive study on the relationship between plasma vitamin D status and the prevalence of asthma in different age groups (e.g., children age 0–17 years), ethnic groups and geographical regions would provide a better understanding of the role that vitamin D may play in the frequency of respiratory infections, and the development/exacerbation of allergies and asthma. Although such a study may confirm or rule out vitamin D, in absence of other plausible leads in the published literature, it may be difficult to design a specific study to determine if there are other possible effects of insolation and UV radiation that could be associated with asthma.

### Conclusions

At 21% for the continental U.S. and up to 43% for the Eastern Seaboard regions, total insolation in winter months is almost as strong as latitude in its ability to explain the observed spatial variation in the prevalence of asthma in the U.S. adult population. Similar results are obtained using the Australian data, suggesting a consistent association between the latitude/insolation and asthma prevalence worldwide.

Taking into consideration confounding factors and possible limitations of an ecological study design, the results presented in this paper suggest that, as a known modulator of the immune response closely linked with geographical latitude and erythemal UV irradiation, vitamin D may play an important role in the development/exacerbation of asthma. Vitamin D is essential for the functional immune system and should be maintained at adequate levels not only in those suffering from asthma but also in the general population. Hence, community-level educational programs on asthma may benefit from including recommendations on adequate diet and vitamin D supplementation to prevent severe deficiencies, particularly among ethnic groups with darker skin pigmentation inhabiting high geographical latitudes.
